# In Silico Study to Identify New Antituberculosis Molecules from Natural Sources by Hierarchical Virtual Screening and Molecular Dynamics Simulations

**DOI:** 10.3390/ph12010036

**Published:** 2019-03-12

**Authors:** Vinícius de S. Pinto, Janay S. C. Araújo, Rai C. Silva, Glauber V. da Costa, Jorddy N. Cruz, Moysés F. De A. Neto, Joaquín M. Campos, Cleydson B. R. Santos, Franco H. A. Leite, Manoelito C. S. Junior

**Affiliations:** 1 Graduate Program in Biotechnology, State University of Feira de Santana, 44036-900 Feira de Santana, BA, Brazil; viniciuspintto@gmail.com (V.d.S.P.); janay@hotmail.com.br (J.S.C.A.); fhenrique@uefs.br (F.H.A.L.); mc2500@gmail.com (M.C.S.J.); 2 Graduate Program in Chemistry, Faculty of Pharmaceutical Sciences of Ribeirão Preto, University of São Paulo, 14040-903 Ribeirão Preto, São Paulo, Brazil; raics@usp.br; 3 Laboratory of Modeling and Computational Chemistry, Department of Biological and Health Sciences, Federal University of Amapá, 68902-280 Macapá, AP, Brazil; vilhenac@hotmail.com; 4 Laboratory of Preparation and Computation of Nanomaterials, Federal University of Pará, 66075-110 Belém, PA, Brazil; jorddynevescruz@gmail.com; 5 Laboratory of Molecular Modeling, State University of Feira de Santana, 44036-900 Feira de Santana, BA, Brazil; moysesfagundes@gmail.com; 6 Department of Pharmaceutical and Organic Chemistry, Faculty of Pharmacy, University of Granada, 18071 Granada, Spain; jmcampos@ugr.es

**Keywords:** virtual screening, pharmacophore model, molecular docking, β-ketoacyl-ACP synthase, tuberculosis

## Abstract

Tuberculosis (TB) is an infection caused by *Mycobacterium tuberculosis*, responsible for 1.5 million documented deaths in 2016. The increase in reported cases of *M. tuberculosis* resistance to the main drugs show the need for the development of new and efficient drugs for better TB control. Based on these facts, this work aimed to use combined in silico techniques for the discovery of potential inhibitors to β-ketoacyl-ACP synthase (MtKasA). Initially compounds from natural sources present in the ZINC database were selected, then filters were sequentially applied by virtual screening, initially with pharmacophoric modeling, and later the selected compounds (based on QFIT scores) were submitted to the DOCK 6.5 program. After recategorization of the variables (QFIT score and GRID score), compounds ZINC35465970 and ZINC31170017 were selected. These compounds showed great hydrophobic contributions and for each established system 100 ns of molecular dynamics simulations were performed and the binding free energy was calculated. ZINC35465970 demonstrated a greater capacity for the KasA enzyme inhibition, with a ΔG_bind_ = −30.90 kcal/mol and ZINC31170017 presented a ΔG_bind_ = −27.49 kcal/mol. These data can be used in other studies that aim at the inhibition of the same biological targets through drugs with a dual action.

## 1. Introduction

Tuberculosis (TB) is a chronic infectious contagious disease that afflicts humanity since ancient times. Despite being preventable and curable, tuberculosis is the ninth leading cause of death worldwide [1]. In 2016, 10.4 million cases and 1.5 million deaths were documented [2].

TB has become a priority for the World Health Organization (WHO) since 1993 [3]. In order to reduce its incidence and mortality, the disease has been linked to the Millennium Development Goals (MDGs). This feature has been proposed by the United Nations, through the global plan for the fight against tuberculosis [4].

The current treatment of tuberculosis presents some negative features, such as a lack of alternatives for the cases of bacterial resistance to the available drugs. Multiple drug-resistant TB occurs when there is resistance to first-line drugs (i.e., rifampicin) and at least one of the second-line injectable drugs [5]. These mechanisms of resistance have contributed to the spread of TB.

The need for new and efficient drugs is evident for better control of TB. Treatment of the disease should not be prolonged, and should not interfere with the administration of antiretroviral agents. The new therapeutic approaches for the treatment of tuberculosis should act on specific targets, without cross-resistance [6,7].

In this scenario, methodologies that can be used to optimize the identification of potential molecules for the TB control are tests in databases [8]. Virtual screening can improve the discovery of potential drug candidates with a reduction in cost and time [9].

Virtual strategies comprise three approaches: The first concerns ligand-based screening, the second is based on the molecular target structure, and finally a combined approach for the first two strategies, pertaining both in the known ligand structures and in the molecular target [10]. Ligand-based screening can be initiated from the knowledge of at least one structure with biological activity. Structure-based design considers the three-dimensional structure of the therapeutic target, using as main strategy the molecular docking simulations for the selection of potential ligands with chemical, electronic and structural characteristics that favor interactions with the molecular target orthosteric site [11].

Mycolic acids and other elements of the mycobacterial cell wall have been proposed as targets in the mechanism of action of drugs used in the treatment of TB. The study of metabolic pathways involved in the biosynthesis of mycolic acids has been used as important steps for the identification of potential targets for the design of new therapeutic agents against this disease [12].

The enzyme β-ketoacyl-ACP synthase (KasA) is a validated and essential target for the survival of mycobacteria. It is therefore an attractive study for the drug development since is based on the Claisen acyl-ACP condensation with malonyl-ACP in the fatty acid synthase pathway in the mycolic acid biosynthesis [13]. Moreover, it is highlighted that KasA is not present in humans, which makes it possible to plan for more selective and safer drugs. The existence of the molecular target along with inhibitors for the target opens up the opportunity to the use of combined virtual techniques, such as screening by pharmacophore models and molecular docking.

After the targeting phase, the next step is the choice of the library of ligands to be explored. Compounds from natural sources are biologically privileged structures, since they have evolved together with proteins during their biosynthesis. Moreover, natural products have a wide and complex chemical diversity, with some properties similar to the drugs in use [14,15]. As reactions in nature are highly influenced by their functions, each natural product generally has a biological receptor and therefore can potentially be a target for drugs. Natural products appear in nature to interact with a given receptor (biomacromolecule), and therefore they could be considered as biologically-validated chemical structures [16].

The objective of the present study is to identify molecules from natural sources through combined virtual strategies for the control of tuberculosis through inhibition of the enzyme KasA. This work is justified mainly by the limited number of alternatives in the treatment of tuberculosis, combined with the high mortality rate, as well as the appearance of tuberculosis resistant cases to current medications.

## 2. Results and Discussion

### 2.1. Selected Compounds that Exhibit Biological Activity with Target

**Figure 1:**
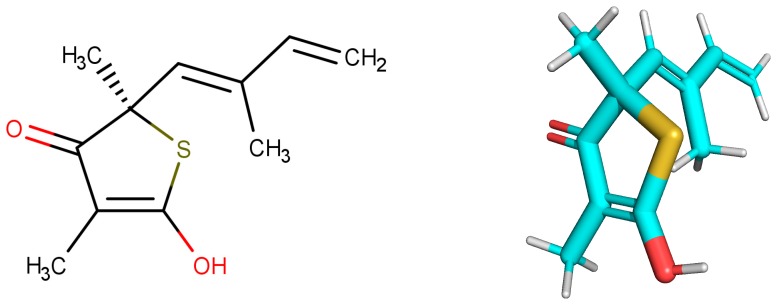
2D and 3D structures of thiolactomycin (TLM).

Thiolactomycin (TLM; Figure 1) is an inhibitor with significant efficacy for MtKasA, but it does not present a considerable inhibition of the other FAS II condensation enzymes, which has led to a greater interest in identifying other potent inhibitors for this target. TLM showed in vivo efficacy in rat models, showing good pharmacokinetic characteristics and antibacterial protection [13].

### 2.2. Construction and Evaluation of Pharmacophore Models

Pharmacophore models are considered the most successful approach in the development of new therapeutic agents, especially since the last two decades [17]. One of the advantages is that this technique allows to prioritize quickly and inexpensively molecules with high potential for interaction with the therapeutic target in large databases [18]. Ten pharmacophore models for MtKasA inhibitors were generated using six molecules of the training set (see Table 1).

From the models generated, only the third model was excluded from the analysis, because they presented an energy value higher than 100.00 kcal/mol, which reflects the difficulty of the molecules to align with the model [19]. Discrepancies between energies between the remaining models can be explained mainly to steric hindrances [20]. The values for Hbond (pharmacophoric concordance) varied from 478.10 to 650.30, while Mol_QRY (fitting of each inhibitor to the model) showed a lower variation from 112.40 to 127.7 kcal/mol.

For the pharmacophore models generated for MtKasA, it was possible to identify that the KasA_002 model (see Table 1) presents the best values for the GALAHAD™ parameters: an energy value equal to 7.53 kcal/mol, Hbond equal to 650.30 and Mol_QRY equal to 112.40.

**Figure 2:**
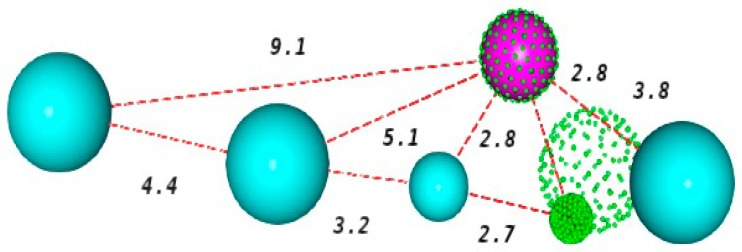
Representation of the best pharmacophore model for KasA inihibitors. Pink: Hbond donor; green: Hbond acceptors; cyan: hydrophobic centers. The size of the spheres represents the tolerance. The distances are shown in angstroms (Å).

Small values for energy and high for Hbond and Mol_QRY are the ideal ones to select the best model [19], because reflects a greater ease of the molecules to mold when aligned with the model, a better pharmacophore agreement and a good fit of each inhibitor to the model. Consequently, such facts have led to the choice of the KasA_002 model. The schematic representation of this model, and the pharmacophore characteristics are shown in Figure 2.

The spheres describe the area that should be occupied by a certain functional group with identical characteristics to those presented by that of the pharmacophore point [21]. The selected pharmacophore model has six characteristics, being two hydrogen acceptor centers (green), one hydrogen donor center (magenta) and four hydrophobic centers (blue).

#### Pharmacophore-Based Virtual Screening

The pharmacophore model displays the key features involved in the interactions within the ligand-target complex. Thereby, this technique can be applied in the quest of compounds, which meet the main molecular requirements for the inhibition of a given target [22].

In order to select candidate molecules for the biological assays against MtKasA, virtual screening was performed based on the pharmacophore characteristics of the selected model. With the total number of compounds present in the ZINC natural-product database (142,788 molecules), 3896 were aligned with the characteristics of the pharmacophore model KasA_002.

The UNITY platform provides a QFIT value as a scoring form for alignment in the model. The value of QFIT can vary from 0 to 100, in which 100 is the best value and presents a greater complementarity with the pharmacophore [23]. The QFIT values for the molecules submitted to screening ranged from 1.47 to 66.76. The five molecules with the best QFIT value are shown in Table 2. ZINC35465970 gives the higher QFIT value for the alignment in the best pharmacophore model of KasA (QFIT = 66.76).

However, the obtained results do not allow the identification of the mode of interaction, molecular volume, as well as do not quantify the affinity energy between the molecule and the receptor, which makes necessary the accomplishment of the docking. Thus, docking assays were performed. The molecules selected for the docking followed the evaluation criterion by the mean value + 2 × standard deviation (34.73). Thereby, 186 molecules were submitted to the docking calculation.

### 2.3. Docking-Based Virtual Screening

Initially, the scoring function was selected. For this, we used some evaluation metrics such as molecular redocking, KISS score calculation, ROC curve analysis and the enrichment factor. The evaluation of the positioning of the ligand in the orthosteric site from the DOCK’s search algorithm was performed through the values of RMSDs, RMSDh and RMSDm. The RMSDs evaluate the deviation between different heavy atom pairs of hydrogen from the reference conformation to the pose after redocking. In turn, the RMSDm is an implementation of RMSD used in AutoDock Vina [24], which does not explicitly consider a mapping atom by atom; the deviation is estimated from the minimum distance between any atom of the same element in reference conformations and recoupled. The RMSDh performs a correction of the symmetry between the heavy atoms [25]. A value of RMSD < 2.0 Å is considered acceptable [26].

**Figure 3:**
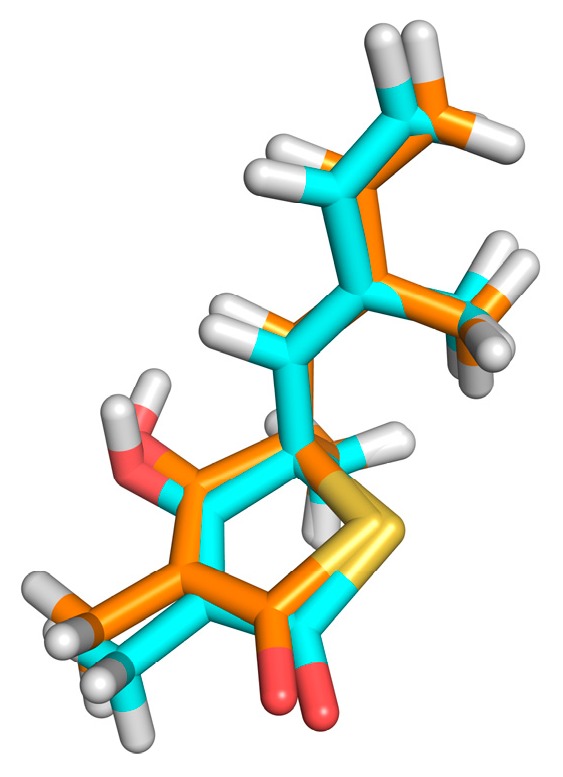
Result of the redocking. Crystallographic ligand in cyan and the best docking pose in orange.

The redocking showed RMSDs = 0.26Å, RMSDh = 0.26Å and RMSDm = 0.14Å (see Figure 3). The methods of calculation of RMSD indicated excellent results, and the DOCK program similarly reproduced the conformation of the crystallographic ligand in the active site of the protein, since the values found were ideal as recommended by literature [11,26]. The second stage of evaluation for the methodology of docking refers to the analysis of the intermolecular interactions after redocking. KISS score is the result of the ratio between the number of hydrogen bonds in the redocked ligand and the number of hydrogen bonds in the crystallographic ligand (PDB = 4C6X). KISS score = 1 was obtained for the target. The interaction of the hydrogen observed with the crystallographic ligand was reproduced after redocking, and no interaction of the same nature was formed. Variations occurred only in the hydrophobic interactions, with the His275 and Phe236 residues.

**Figure 4:**
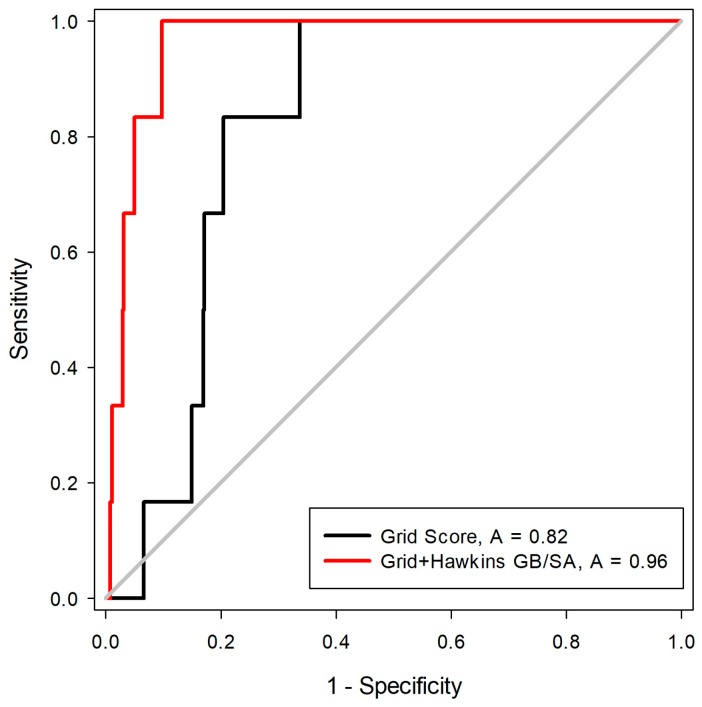
ROC curves for evaluation of Grid-Hawkins GB/SA and Grid Score.

The third stage for the analysis of the scoring functions consisted in the evaluation of the Receiver Operating Characteristic (ROC) curve. This step is one of the best ways to compare the performance of scoring functions and classifiers [27]. For this, a library was constructed with 550 decoys and six inhibitors, five of these with two states of protonation. After the construction of the molecule bank (ligands + decoys), the docking tests were carried out with two scoring functions (Grid-Hawkins GB/SA and Grid Score). The graph with the ROC curve with the two scoring functions applied can be visualized in Figure 4.

From the ROC curve graph, it was possible to evaluate the performance of scalar measures of classification, as specificity and sensitivity. The ROC curve is a graphical representation of the sensitivity (proportion of true positives) as a function of specificity (proportion of false positives). The value of the area on the curve (AUC) provides an objective measure of the overall performance of a classifier. An AUC value equal to 1 (or 100%) indicates that the active and inactive compounds are perfectly discriminated, while a value of 0.5 (or 50%) is understood as a random performance [28]. Matsubara’s studies [29] show that, in general, the accuracy of the classification method can be evaluated with the following scale: 0.9–1: excellent; 0.8–0.89: good; 0.6–0.79: reasonable; 0.5–0.59: poor; and below 0.49 corresponds to a complete failure.

From the analysis of the ROC curve, it was possible to observe that the scoring function proved to be the most efficient in the recovery of the bioactive compounds for MtKasA was Grid + Hawkins GB/SA, with an AUC = 0.96, which can be considered as an excellent value for the classification.

**Figure 5:**
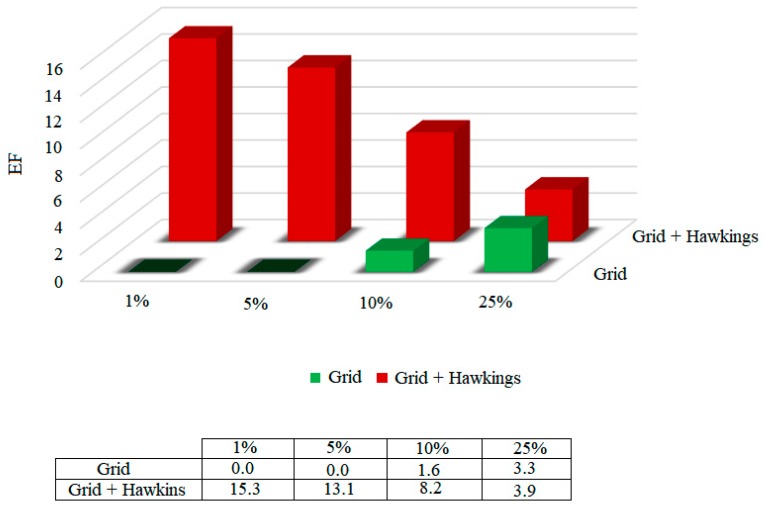
Enrichment factor (EF) for scoring functions used in MtKasA in 1, 5, 10 and 25% of the database.

One of the problems pointed out to the AUC value is the fact that it is a global measure, not clearly presenting information about the early recognition of active compounds [30,31]. On the other hand, the enrichment factor (EF) quantifies the proportion of active compounds identified when analyzed in a given proportion (nX%) of the total set of ordered compounds. The enrichment factors were calculated for the functions, considering 1%, 5%, 10% and 25% of the bank that was coupled to. The results are shown in Figure 5.

According to the results presented in Figure 5, it was possible to highlight that, among the two scoring functions tested, the Grid + Hawkins GB/SA function presents a 15.3 times greater chance of identifying the active compounds in only 1% of the database.

Considering the two analyzes, the area on the curve and the enrichment factor, the Grid + Hawkins GB/SA scoring function was presented as the methodology that best classifies, and therefore presents the greatest accuracy.

**Figure 6:**
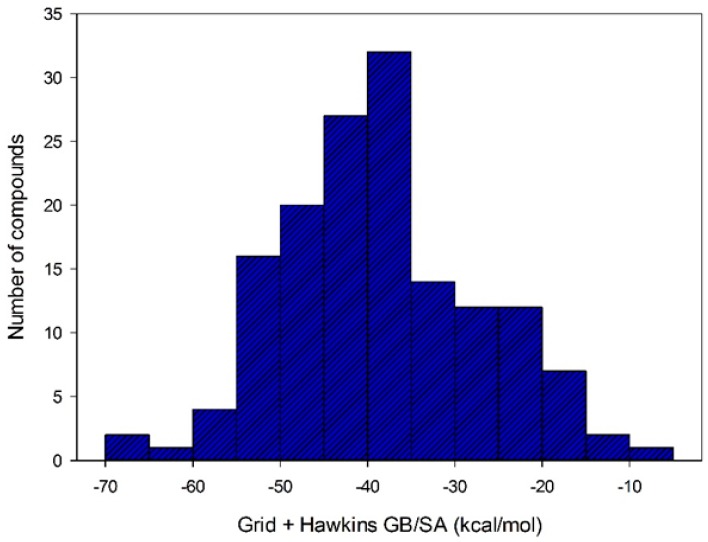
Distribution of compounds according to their affinity energy against MtKasA.

After selecting the scoring function with greater accuracy for each target, the virtual screening was performed by docking using structures with a QFIT value equal to or greater than 34.73. Out the 186 molecules subjected to the docking, only 152 achieved some affinity to MtKasA. The affinity energy values for these molecules ranged from −4.87 to −67.70 kcal/mol (see Figure 6).

In an attempt to relate the QFIT values obtained from similarity screening and the affinity energy values resulting from docking calculations, a consensual analysis was performed using these two variables (see Table 3).

The structure with the best score for MtbKasA was ZINC35465970, which has 332.5 g/mol of molecular mass, three Hbond acceptors, three Hbond donors, nine rotatable bonds and partition coefficient (logP) of 6.44. Information extracted from ZINC [32] PubChem [33] and ChemSpyder [34] indicate that such a molecule has not been subjected to biological evaluation. ZINC31170017 presented the second best value of consensus, and the analysis of the physical-chemical properties indicates that this molecule has 462.5 g/mol of molecular mass, nine Hbond acceptors, six Hbond donors, ten rotatable bonds and 1.02 xlogP. It was not possible to identify experiments related with the biological activity for ZINC31170017. Therefore, from the analysis of the scoring function and the similarity alignment (QFIT), molecules ZINC35465970 and ZINC31170017 were submitted for analysis of the intermolecular interactions.

**Figure 7:**
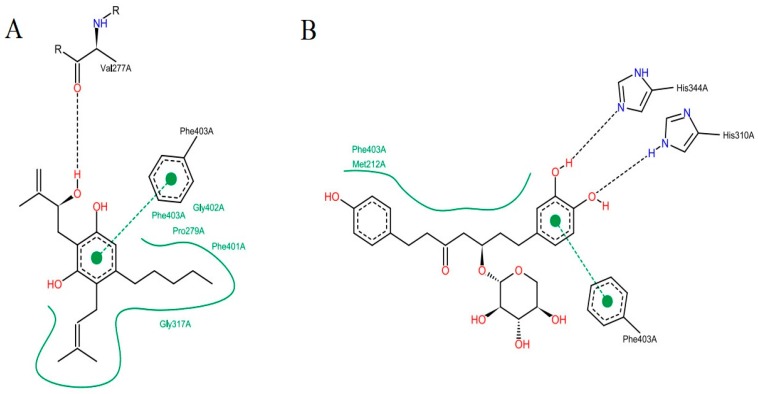
Interactions of compounds against the active site of *Mycobacterium tuberculosis* KasA, In (**A**) ZINC35465970 and (**B**) ZINC31170017.

The analysis of the intermolecular interactions is useful for identification and optimization of contacts between ligands and target [35]. The results for the two molecules selected are shown in Figure 7: It is possible to point out a greater contribution of the hydrophobic interactions in both molecules. ZINC35465970 (Figure 7A) performs hydrophobic interactions of its aliphatic chains attached to the dihydroxybenzene ring with the Phe403, Pro 279, Phe401, Gly402 and Gly317 residues. In addition to these interactions, this molecule forms aromatic interaction π-π, between the dihydroxybenzene ring and the aromatic ring of the side chain of Phe403. It is also possible to observe a hydrogen interaction between a hydroxyl of the molecule with the Val277 ketone.

Considering the complex formed between compound ZINC31170017 (Figure 7B) and MtbKasA, hydrophobic interactions with Phe403 and Met212 residues can be observed. In addition, a hydrophobic π-π interaction with Phe403 and the dihydroxy benzene ring of the molecule can be observed, also present in the molecule with the best score, indicating that this residue may be involved in the molecular recognition process. The third type of binding present is a hydrogen interaction, occurring between the two hydroxyls of the dihydroxy benzene ring with the imidazole rings of His344 and His310. Hydrogen interactions with the same residues (His310 and His344) have already been verified in other studies [36].

### 2.4. Structural Analysis of Systems

**Figure 8:**
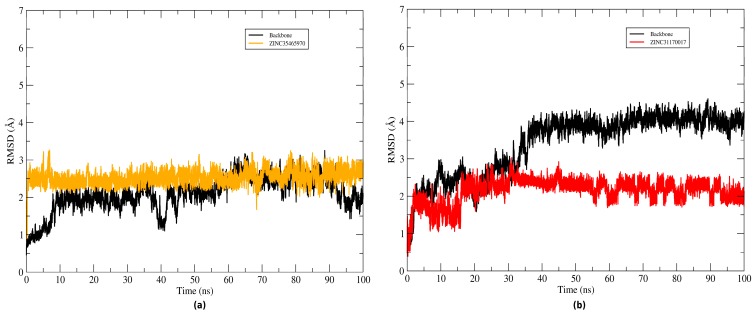
Evaluation of RMSD plots during MD simulations. The KasA protein backbone has been represented in black, while the ligands graphs have been represented in different colors. (**a**) RMSDs of the KasA- ZINC35465970 system and (**b**) RMSDs of the KasA-ZINC31170017 system.

The root mean square deviation (RMSD) of the atomic positions of protein was plotted to evaluate the structural stability of the complexes (KasA-ZINC31170017 and KasA-ZINC35465970) and the binder backbone along the molecular dynamics trajectory. To plot the backbone and RMSD of the complex graphs the Cα atoms and heavy atoms were used, respectively. The graphs plotted of systems can be seen in Figure 8. Throughout the simulation time, the inhibitors remained bound to the active site of the protein. The ZINC35465970 ligand remained in equilibrium exhibiting slight structural divergences. During approximately the 20 ns of the initial simulation, ZINC31170017 underwent several conformational changes, but along the trajectory reached a balance and started to exhibit small changes in KasA conformation. Thus, the complexes were considered stable and not discarded for a further analysis.

**Figure 9:**
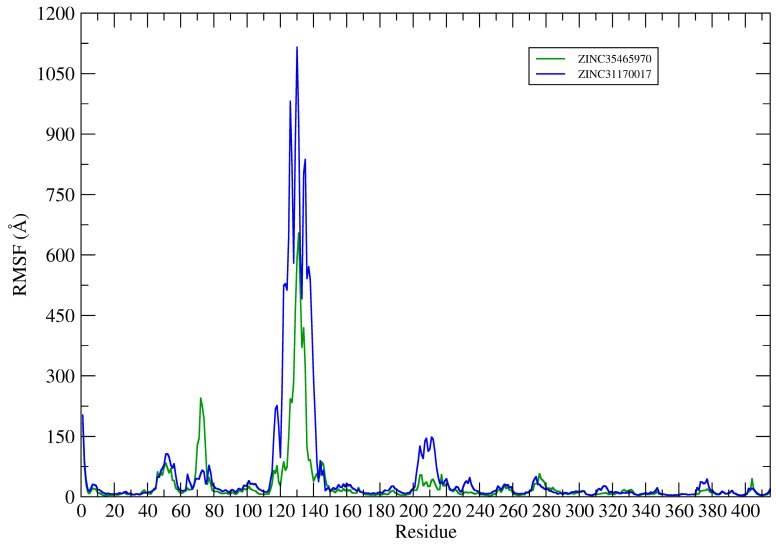
Protein backbone RMSF plots.

This way, the root mean square fluctuation (RMSF) of the complexes residues (KasA-ZINC31170017 and KasA-ZINC35465970) have been plotted using the Cα atoms to analyze the protein backbone (see Figure 9).

**Figure 10:**
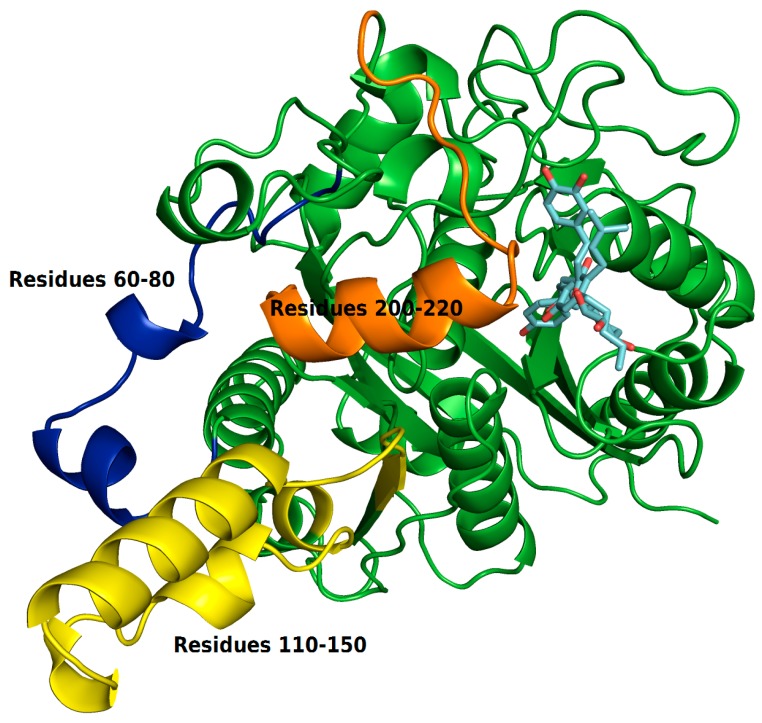
Regions of protein that showed greater residue fluctuations.

The RMSF plots revealed differences in protein flexibility throughout the trajectory. The largest differences in residue fluctuations occurred in the range of residues 60 to 80, 110 to 150 and 200 to 220. In Figure 10 these protein segments were colored and identified.

The amino acids 60 to 80 form a loop region and two small alpha helices that are exposed to the solvent. The fluctuation of this backbone region may be related to structural features of loop regions that naturally have a degree of flexibility.

ZINC35465970 compound shifted from its molecular docking position further into the bonding cavity during the simulation. In the molecular docking pose, this ligand did not show many interactions with the alpha helix residues and the loop comprised between residues 200–220. However, after MD simulation and with the balance of ligand in the binding pocket, two hydrophobic chains of the complex underwent conformational reorientation, thus establishing greater hydrophobic interactions with the residues in the range of 200 to 220. ZINC31170017 showed interactions with few residues present at the beginning of alpha helix formed by the residues in the range that we have analyzed. In this way, the alpha helix region remained freer to move, and consequently demonstrating a greater fluctuation.

The region of the protein that showed the greatest fluctuation is composed by the residues 110 to 150. In the system established with ZINC31170017 this region presented a more open conformation, more exposed to the solvent, whereas the same region of the protein in the system formed with ZINC35465970 remained in a tightest conformation, in comparison to each other.

#### 2.4.1. Hydrogen Bonds Established between Receptor-Ligands

Taking the RMSD and RMSF results are a visual analysis of representative MD trajectory, the formation of hydrogen bonds during the entire computational simulation time was carried out to investigate the interaction profile of complexes. The main interactions established are shown in Table 4.

There was a difference between the number of hydrogen bonds established by compounds in the binding pocket. ZINC35465970 showed the highest number of hydrogen bonds formed, which can justify its greater structural stability verified in the RMSD graph and also its greater capacity to connect the enzyme, and consequently its greater power of inhibition verified in binding free energy value (ΔG_bind_ = −30.90 kcal/mol). However, ZINC31170017 established only two hydrogen bonds with the receptor, with this presented lower conformational stability throughout the simulation and less capacity of interaction with the protein.

#### 2.4.2. Bind Free Energy KasA-Ligands

The free energy values and their energy components are summarized in Table 5. The results obtained by the MM/GBSA method suggest that ZINC35465970 has a greater capacity to inhibit the KasA enzyme, since we obtained the value of ΔG_bind_ = −30.90 kcal/mol, whereas the ZINC31170017 reached the value of ΔG_bind_ = −27.49 kcal/mol. The van der Waals interactions (ΔE_vdW_) were the main responsible for maintaining the enzyme-inhibitor complexes, in the systems formed, ΔE_vdW_ presented values of −45.21 kcal/mol and −35.86 kcal/mol, to ZINC35465970 and ZINC31170017, respectively. The electrostatic (ΔE_ele_) and non-polar (ΔG_NP_) contributions also favored the established systems. For the interaction with ZINC35465970, ΔE_ele_ = −11.93 kcal/mol and ΔG_NP_ = −6.06 kcal/mol for ZINC31170017 ΔE_ele_ = −11.48 kcal/mol and ΔGNP = −5.18 kcal/mol were obtained.

## 3. Materials and Methods

### 3.1. Dataset

Initially a dataset with potent KasA inhibitors (Ki < 40 µM) [36] was employed for construction and evaluation for pharmacophore modelling (Table 6). The subset of natural products from the ZINC database (https://zinc.docking.org) [32] with 142.788 compounds were selected for virtual screening. All molecules were drawn on the Marvin Sketch software [37]. Then they were prepared through calculation of the Gasteiger-Hückel charges and addition of the hydrogen atoms using the DockPrep module implemented at Chimera 1.10.1 [38].

### 3.2. Pharmacophore Models Construction

Pharmacophore models were generated through the GALAHAD module, available on the SYBYL-X 2.0 platform [39]. For the generation of pharmacophore models, the training set (*n* = 6) was aligned based on its pharmacophore characteristics to produce the respective models using default genetic algorithm parameters (Cross-over = 1.0 and Mutation = 0.4). 

The alignment process was conducted in two steps: in the first, the selected compounds were aligned mutually in a given spatial coordinate using an advanced genetic algorithm (GA) to allow flexibility of the molecules. In the second step, the molecular alignment was based on the overlapping of its pharmacophore characteristics in the conformations generated by the GA of the compounds in the Cartesian space. Thus, to achieve a hyper molecular alignment, the population size and maximum generation of GA simulation were set at 40 and 45, respectively. The other genetic operators were maintained as the default configuration, as established in SYBYL-X 2.0.

The pharmacophore models were evaluated based on the statistical parameters of GALAHAD [39]. These parameters include Hbond, Mol_Qry, and energy. First, models with strain energy, two orders of magnitudes above the others, were discarded. Next, the pharmacophore models were analyzed based on the value of Hbond and Mol_QRY, that one with best result based on the sum of these two parameters was selected for virtual screening.

#### Pharmacophore-Based Virtual Screening

The model selected with the best requirements was employed to screen the natural products in the ZINC database through UNITY 3D implemented on SYBYL X 2.0 [39]. These molecules were evaluated by QFIT value. Molecules with QFIT = 0 were discarded.

Thus, the compounds were staggered according to the QFIT value, and the molecules were selected for the docking-based on the cut-off point (COP) obtained by the following Equation (1):

*PdC* = ±2σ (1)

### 3.3. Docking-Based Virtual Screening

The crystallographic structure was selected in the Protein Data Bank (PDB ID 4C6X). The 3D structure was treated with the DockPrep implemented on Chimera 1.9 [37], thereby the hydrogen atoms were added and the Gasteiger partial atomic charges (ff12SB) were attributed to the protein residues [40]. The PDB2PQR program [41] was employed to calculate the protonation state.

Docking was carried out in DOCK 6.5 [25]. Through molecular surface to access receiver solvent and the negative image of the orthosteric sites were generated by the accessory program DMS [42], SPHGEN and SPHERE_SELECTOR programs respectively [43]. Properties were calculated by the GRID program in its standard configuration [44,45]. The following scoring functions were used: GridScore and GridScore + Hawkings GB/SA.

The evaluation step of the scoring functions contemplated some different methodologies. First, one was the calculation of the mean square deviation (RMSD) obtained by the two scoring functions in relation to the repositioning of the crystallographic ligand. Then, the Key Interaction Score System (KISS) score [46] was calculated, in which the hydrogen bonds are evaluated after the redocking process. KISS scores were obtained using Equation (2) below [46]:

KISS score = *I_r_/I_n_* (2)

where, *I_r_* represents the amount of hydrogen bonds carried out between the best posture of the coupled ligand and I_n_ is the total of hydrogen bonds present between the crystallographic structure of the ligand and the target.

The scoring function with RMSD ≤ 2.0Å were evaluated by ROC curve performance and AUC value based on protocol established by our research group [47]. In order to analyze the recovery rate of true positive inhibitors, it was necessary to generate false-positive molecules (decoys) with DUD-E server (http://dude.docking.org) [48], using same training set employed on pharmacophore modelling (Table 1). The data were plotted for Receiver Operating Characteristic (ROC) construction and later Area Under the Curve (AUC) analysis. The enrichment factor was also used to complement the evaluation of the scoring functions.

From the selection of the most sensitive scoring function in the recovery of the active molecules, the molecules that were selected according to the QFIT scores in the alignment step in the pharmacophore model were submitted to docking. After docking, the molecules were arranged in order of the affinity estimated against KasA.

In order to correlate the results obtained by the docking calculations with the results of the filtering by pharmacophore modeling, we also adopted the number-by-number classification strategy with the application of variable recategorization, which combines the results of the strategies used [49]. The scores obtained for the QFIT value and energy values were summed up, resulting in an overall score for each molecule. The analysis of intermolecular interactions and 2D intermolecular plots were built up on the PoseView web 1.97.0 server [49,50].

### 3.4. Molecular Dynamics (MD) Simulations

The atomic charges of the ligands were obtained by performing the Restrained Electrostatic Potential (RESP) using HF/6-31G * [51]. Then the parameters of the ligands were generated with the Antechamber module being described by General Amber Force Field (GAFF) [52], whereas the force field ff12SB [53] was applied to treat the proteins. To neutralize the overall partial load of the systems sodium ions were added, soon after the complexes were solvated in a truncated octahedral water box with periodontal conditions and water molecules described by the TIP3P model [54].

For the energy minimization calculations of the system the sander.MPI, and for the heating steps and molecular dynamics the pmemd.CUDA of the Amber 16 package were used [55,56].

The energy of the systems was mimicked in five stages where in each one were executed 3000 cycles using the steepest descent method and 5000 cycles using the conjugate gradient algorithm. In the first stage the hydrogen of the water molecules were minimized, and in the second stage the water molecules and ions, soon after the hydrogen atoms of the protein and finally, all the solvent and solute had their energy minimized. The systems were heated gradually to 300 K for 650 ps and was performed using NVT ensemble. The collision frequency used was 3.0 ps^−1^ and the Langevin thermostat was used for temperature control [57]. After warming up, 2 ns of MD simulations using NPT ensemble were run to balance the system. Lastly, for each system 100 ns of MD simulations were generated. The Particle Mesh Ewald method was used to calculate the electrostatic interactions [58] and the bonds involving hydrogen atoms were restricted with the SHAKE algorithm [59].

#### Binding Free Energy Calculations

To estimate the binding free energy of the KasA protein with the ligands ZINC35465970 and ZINC31170017 we used the Molecular Mechanics/Generalized Born Surface Area (MM/GBSA) method [60,61]. For our calculations, we used 500 snapshots of the last 5 ns of MD simulation.

The free energy was estimated according to Equation (3):

ΔG_bind_ = ΔE_MM_ + ΔG_solv_ − TΔS (3)

ΔG_bind_ is the affinity energy resulting from the sum of the total energy in the gas phase (ΔE_MM_), free energy of solvation (ΔG_solv_) and entropy (TΔS).

ΔE_MM_ is the sum of ΔE_internal_ (connections, angles and dihedra), ΔE_electrostatic_ (electrostatic contributions) and ΔE_vdW_ (Van der Waals contributions), according to Equation (4):

ΔE_MM_ = ΔE_internal_ + ΔE_electrostatic_ + ΔE_vdw_ (4)

ΔG_solv_ can be obtained by solving Equation (5):

ΔG_solv_ = ΔG_GB_ + ΔG_SASA_ (5)

When the polar contributions (ΔG_GB_) are calculated using either the GB model or the non-polar contributions (ΔG_SASA_), they were determined from the calculation of the solvent accessible surface area (SASA).

## 4. Conclusions

Ligand-based and target-based techniques have been useful in identifying potential inhibitors. In this sense, this work applied these two approaches through a pharmacophore model and molecular docking studies. The pharmacophore modeling strategy resulted in the selection of a model with acceptable statistical parameters. The molecules filtered by the model show the physicochemical properties of inhibitors known for the MtbKasA. These molecules were evaluated through molecular docking. Through these results, a consensual evaluation was made that provided the prioritization of molecules. The two best molecules scored were analyzed for the interaction mode with the target, which presented π-π interactions, hydrogen bonds and hydrophobic interactions. We also performed MD simulations by which we evaluated the structural stability of the systems and observed that certain regions of the protein showed differences in their flexibility when interacting with the inhibitors along the simulation trajectory. We also verified by means of free energy calculations with the MM/GBSA method that the inhibitor ZINC35465970 was that presented better ability to inhibit the target KasA (ΔG_bind_ = −30.90 kcal/mol) and that the interactions of the van der Waals type were the main responsible for the formation of the systems.

This work allowed the identification of possible inhibitors of MtKasA, which is an essential phase in the design of new drugs. It is necessary to apply in vitro and in vivo assays to confirm the biological potential of these molecules.

## Figures and Tables

**Table 1:** Parameters of GALAHAD™ for models from MtKasA inhibitors. The pharmacophore model that presented an energy penalty is indicated by the red color.

Models	Strain Energy (kcal/mol)	Hbond	Mol-qry
2	7.53	650.30	112.40
8	7.58	613.30	114.60
1	8.02	619.30	127.70
5	9.05	497.20	122.60
10	9.54	478.10	125.10
4	10.52	520.90	128.40
7	45.88	504.00	126.20
9	67.80	550.70	132.00
6	69.55	482.60	136.70
3	496.79	655.80	133.10

**Table 2:** The five molecules with the best QFIT value.

Molecule	Structure	QFIT
ZINC35465970	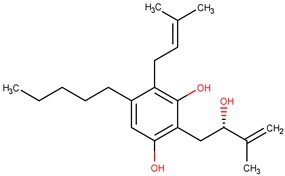	66.76
ZINC15959689	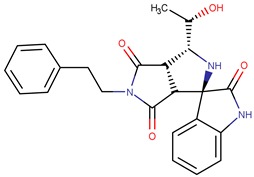	62.97
ZINC16032930	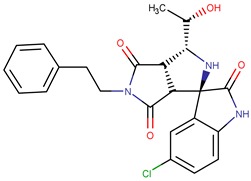	62.07
ZINC31161132	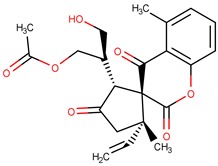	59.99
ZINC72320274	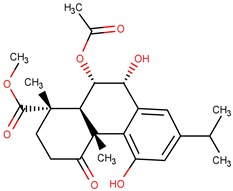	59.86

**Table 3:** Consensus ranking of variables: relationship between the result of QFIT and Grid + Hawkins GB/SA for MtbKasA.

MOLECULE	CONSENSUS
ZINC35465970	122.10
ZINC31170017	108.57
ZINC12659549	108.52
ZINC08453820	107.44
ZINC15959689	107.28

**Table 4:** Hydrogen bonds formed in complexes.

Acceptor	Hydrogen Donor	Donor	Occupancy (%) ^a^	Average Distance (Å)
**ZINC35465970**				
ZINC35465970_416@O25	GLN_170@HE22	GLN_170@NE2	30.46	3.17
ZINC35465970_416@O25	HIS_344@HE2	HIS_344@NE2	28.75	3.03
ZINC35465970_416@O24	LYS_339@HZ3	LYS_339@NZ	21.69	2.89
ZINC35465970_416@O24	LYS_339@HZ1	LYS_339@NZ	21.19	2.89
ZINC35465970_416@O24	LYS_339@HZ2	LYS_339@NZ	20.01	2.90
**ZINC31170017**				
MET_212@O	ZINC31170017_416@H62	ZINC31170017_416@O37	71.27	2.81
ARG_233@O	ZINC31170017_416@H56	ZINC31170017_416@O22	49.69	3.01

**^a^** Occupancy is defined as the percentage of time that hydrogen bonding existed during the 100 ns simulation time.

**Table 5:** Energy contributions to the free energy binding KasA-compounds.

Compound	ΔE_vdW_	ΔE_ele_	ΔG_GB_	ΔG_NP_	ΔG_bind_
ZINC35465970	−45.21	−11.93	32.31	−6.06	−30.90
ZINC31170017	−35.86	−11.48	25.04	−5.18	−27.49

**Table 6:** KasA inhibitors selected for construction and evaluation of pharmacophore models.

Molecule	Structure	Ki (µM)
1	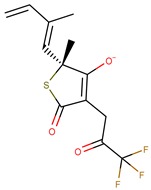	0.46
2	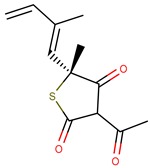	0.90
3	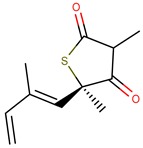	1.90
4	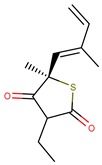	7.10
5	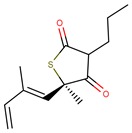	16.00
6	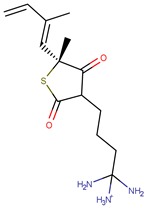	34.00
